# Gloss, Colour and Grip: Multifunctional Epidermal Cell Shapes in Bee- and Bird-Pollinated Flowers

**DOI:** 10.1371/journal.pone.0112013

**Published:** 2014-11-04

**Authors:** Sarah Papiorek, Robert R. Junker, Klaus Lunau

**Affiliations:** 1 Institute of Sensory Ecology, Department Biology, Heinrich-Heine University Düsseldorf, Düsseldorf, Germany; 2 Department of Organismic Biology, University Salzburg, Salzburg, Austria; University of Sussex, United Kingdom

## Abstract

Flowers bear the function of filters supporting the attraction of pollinators as well as the deterrence of floral antagonists. The effect of epidermal cell shape on the visual display and tactile properties of flowers has been evaluated only recently. In this study we quantitatively measured epidermal cell shape, gloss and spectral reflectance of flowers pollinated by either bees or birds testing three hypotheses: The first two hypotheses imply that bee-pollinated flowers might benefit from rough surfaces on visually-active parts produced by conical epidermal cells, as they may enhance the colour signal of flowers as well as the grip on flowers for bees. In contrast, bird-pollinated flowers might benefit from flat surfaces produced by flat epidermal cells, by avoiding frequent visitation from non-pollinating bees due to a reduced colour signal, as birds do not rely on specific colour parameters while foraging. Moreover, flat petal surfaces in bird-pollinated flowers may hamper grip for bees that do not touch anthers and stigmas while consuming nectar and thus, are considered as nectar thieves. Beside this, the third hypothesis implies that those flower parts which are vulnerable to nectar robbing of bee- as well as bird-pollinated flowers benefit from flat epidermal cells, hampering grip for nectar robbing bees. Our comparative data show in fact that conical epidermal cells are restricted to visually-active parts of bee-pollinated flowers, whereas robbing-sensitive parts of bee-pollinated as well as the entire floral surface of bird-pollinated flowers possess on average flat epidermal cells. However, direct correlations between epidermal cell shape and colour parameters have not been found. Our results together with published experimental studies show that epidermal cell shape as a largely neglected flower trait might act as an important feature in pollinator attraction and avoidance of antagonists, and thus may contribute to the partitioning of flower-visitors.

## Introduction

Plant-animal interactions include mutualistic as well as antagonistic relationships. Animal pollination was traditionally regarded as mutualism including reciprocal benefits for both interaction partners. Nowadays the view that the flowers' signalling apparatus single task is the attraction of flower visitors has changed. Flowers are interpreted as sensorial and/or morphological filters supporting the attraction of pollinators as well as the deterrence of floral antagonists such as herbivores, pollen and nectar robbers or thieves (reviewed in [1]). These interactions bear on different communication tasks, with colour as one of the most important floral features that structures the flower-visitor composition [2], [3]. The diversity of flower colours in angiosperms is mainly attributed to pigments deriving from different biochemical pathways, their combinations, variable concentrations as well as additional co-pigments, the prevalent pH in the vacuole, metal ions, pigment packaging and location within the tissue layers [4]. Next to these factors, also the petals' epidermal cell structure affects the visual appearance of flowers [5], [6], [7], [8], [9]. Particularly, conical epidermal cells can act as lenses and light traps, changing optical properties by refracting and focusing light into the pigment containing tissue layer of petals [5]. Gorton & Vogelman [5] investigated this function in the Snapdragon *Antirrhinum majus*, whose wild type flowers have conical epidermal cells and a comparably enhanced colour signal. By contrast, mutants with flat instead of conical epidermal cells are focusing incident light into the mesophyll beneath the pigment-containing epidermal cell layers, thereby reducing the colour signal as the pigments absorb comparably less light [5]. Thus, the presence of conical epidermal cells in contrast to flat ones might alter colour impression for flower-visitors by enhancing light absorption by pigments in a yet unexplored manner [10].

Moreover, the structure of epidermal cells affects the amount of gloss at the petals' surface. Gloss is defined by the total reflectance of incident light at a surface in the identical angle to that of the incident light. Both, in theory and as shown in experimental studies on flowers [8], [9], [11], gloss is strongest if the surface is flat, in this case, if the epidermal cells are flat. Thus, at smooth surfaces a smaller portion of the incident light enters the plant tissues and passes the pigment containing cells as compared to rough surfaces. With decreasing reflectance at mirror geometry (i.e. gloss), the colour signal increases, as a higher amount of light enters the tissue and might be absorbed by pigments. Gloss is a phenomenon appearing in fruits and petals of some plant species [8], [9], [12], [13], [14], [15], but the vast majority of angiosperm flowers exhibits some form of conical epidermal cells [11] and possesses only slightly glossy surfaces. Absorption of light by flower pigments causes a spectral signal restricted to specific ranges of wavelengths, whereas gloss phenomena cover the whole range of visible light, including the ultraviolet range of wavelengths, and are strictly angle-dependent [14].

Beside visual appearance, the epidermal cell structure also determines floral temperature [16], floral shape [6], wettability [17], microsculptural patterns forming nectar guides [18], and floral grip [19], [20], [21]. The latter involves that conical epidermal cells provide contact between bee and flower petal, making flowers easier to handle as they are less slippery [19].

In the current study we investigate the shape of epidermal cells on flowers pollinated by either bees or birds. Due to differences in the visual capabilities as well as differences in the foraging behaviour between these two flower-visitor groups, differences in respect to epidermal cell shapes and their consequent functions are conceivable. The colour vision system of bees and flower-visiting birds differ in respect to the number of photoreceptor types, with superior colour discrimination abilities in birds as compared to bees [22], [23], [24]. Colour is an important trait for foraging bees to detect flowers evoked by innate colour preferences and learned responses ([25], and references within), whereas flower-visiting birds do not show preferences for specific colour properties, but nevertheless associate colours with floral rewards [26], [27]. Thus, we investigate three different hypotheses concerning the effects of epidermal cell shape on 1) the colour sensation for bees as well as birds, 2) the importance of floral grip for bees and birds, and 3) on floral grip for nectar robbers.

The first hypothesis implies that bee-pollinated flowers might benefit from enhancing the flower's colour signal for bees, whereas bird-pollinated flowers benefit from avoiding frequent visits by bees due to flower colours which are comparatively less attractive to bees [2]. As bird-pollinated flowers become commonly thieved by bees representing competitors for pollinators, negative effects on the plants' fitness arise [28], [29], and avoidance of frequent visitation by bees should be beneficial for these plant species [2]. Unlike conical epidermal cells, flat ones may bear that function by producing less attractive colours for bees with similar pigment concentrations.

The second hypothesis based on the knowledge that bees need grip to effectively forage on flowers [19], [20]. Again, bee-pollinated flowers might benefit from conical cells promoting floral grip. In contrast, bird-pollinated flowers might benefit from flat epidermal cells, hampering bees from effective foraging and thus, avoiding the loss of rewards for their pollinators.

The third hypothesis implies that those flower parts vulnerable to nectar robbing benefit from flat epidermal surfaces, hampering grip and handling for bees while robbing the flower [19], [20]. Since this parameter seems relevant for bee- as well as bird-flowers we investigate whether robbing-sensitive parts possess mechanical properties, i.e. flat epidermal cell surfaces, which may help to avoid nectar robbing.

Testing these three hypotheses allows us to demonstrate that epidermal cell shape is a multifunctional flower trait which may act as an important feature in pollinator attraction.

## Materials and Methods

### Plant material

In total we studied the epidermal cell shape, spectral reflectance and gloss of the flowers of 58 plant species from 48 genera in 26 families (29 species from 28 genera in 16 families which are adapted to the pollination by bees, and 29 species from 23 genera in 15 families adapted to the pollination by birds, Text S1). Flowers were collected in the Botanical Garden of the Heinrich-Heine University, Düsseldorf, Germany. The permission for collecting three to five flowers was obtained by the academic advisor Dr. Sabine Etges. Flowers were stored in moist storage boxes until measurements as soon as possible after picking the flowers at the same day.

Plant species were categorized into bee- and bird-pollination, with effective pollinators assigned from literature (Text S1). For this purpose, plant species were only included, if literature reports seed or fruit set caused by specific flower-visitors or if literature explicitly supports morphological fit between frequent visitors and the flowers' reproductive organs, as evidence for effective pollination. To circumvent incorrectly assigned pollinators from our data set, we excluded those plant species from our analysis for which only assumptions of probable pollinators were made from morphological floral traits according to pollination syndromes [30]. For example, the red flower colour is often assigned with bird-pollination, but bees can contribute to effective pollen transfer, performing selection pressure towards flower traits promoting visitation by bees [31], [32].

Flower parts were categorized into those sites which belong to the visually signalling apparatus of the flower and provide a landing platform for bees (afterwards referred to as ‘visually-active parts’) and those that are averted from the visitor and are vulnerable to nectar robbing by bees (afterwards referred to as ‘robbing-sensitive parts’) (Text S1). In tubular flowers the former parts were adaxial parts of lips and the latter ones abaxial basal parts of the corolla; in open flowers the former parts were adaxial parts of petals and the latter ones abaxial basal parts of the petals (Text S1).

### Shape measurements

**Figure 1:**
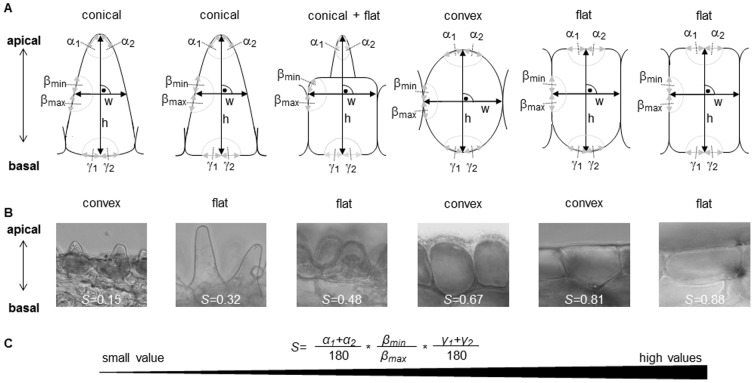
Cell shapes and explanation of the shape index *S*. A) Drawings and B) photographs of epidermal cell shapes with *S*-values for the represented shape types (from left to right: *Chritia gueilinensis*, *Proboscidea fragrans*, *Tecomaria capensis*, *Columnea gloriosa*, *Aloe vogtsii*, *Polygala myrtifolia*), found in the epidermal surfaces of investigated flowers. h = maximal cell height. w = cell width at half height. Dashed grey curves indicate angles. Grey arrows indicate a length of 5 µm. C) Formula of shape index *S*.

Several types of epidermal cells can be found in flower petals [11], with six main types comprised in our data set (Figure 1A, C). Light that already entered the epidermal cell tissue of petals will be reflected from the underlying mesophyll towards the outside and thereby passing the epidermal cells again [11]. If the basal epidermal cell parts are convex or conical, and the refractive indices differ between the epidermal cell and the mesophyll cell, light might be focused again into the pigment containing area. Thus, we assumed that the apical as well as basal part of the epidermal cells contribute to light refraction. Therefore we defined a shape index *S* from three angles, i.e. α, β, and γ, describing the degree of bending of the surface of the apical, lateral as well as the basal cell part:
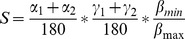
(1)Cell shape was characterized by means of transverse sections of petals using light microscopy. Slices were made at the same parts and in the same direction, where spectral reflection and floral gloss measurements were taken, as the arrangement of epidermal cells may vary with respect to their position on the petal and as microstructural pattern can differ in various parts of petals [18]. Slices had a thickness of three to four cell-rows and we focused on the inner cell-row in order to assess the cell morphology without any underestimation of cell parameters, especially cone steepness. Those slices where cells were only cut were not considered. LM-photographs were analysed using AxioVision Rel. 4.8 Software (ZEISS, Oberkochen, Germany) by evaluating the maximal cell height (h) and the cell width orthogonal to half the maximal cell height (w; Figure 1A).

Each angle is located between a length parameter, i.e. h or w, and the adjacent cell wall in a distance of 5 µm from the intercept of cell wall and length parameter. α_1_ and α_2_ describe the surface structure in the apical part of the epidermal cell (Figure 1A). The smaller α_1_ and α_2_, the more conical, the larger α_1_ and α_2_ the more flat is the cell towards the petal outside (Figure 1B). The same is true for γ_1_ and γ_1_, describing the bending of the cells' basal part (Figure 1A, B). The ratio between β_min_ and β_max_ describe the degree of bending of the lateral cell part towards the adjacent epidermal cells (Figure 1A). This term was added in order to include those cell forms, which consist of a flat part with attached papillae, forming a cell form with flat as well as conical parts and therefor intermediate *S*-values (Figure 1B).

For each flower part of each flower we evaluated the mean values of each shape parameter of five haphazardly chosen cells and afterwards calculated *S*. In summary, the smaller *S*, the more conical is the cell towards the apical and basal part and the higher *S*, the more flat is the cell form towards each side (Figure 1B). Reverse-conical cells for example have intermediate values for *S* (Figure 1B). *S* values for all investigated flower parts of all plant species are given in Text S1.

### Gloss measurements

Gloss measurements were made using a ZGM 1120 Glossmeter (Zehnter Testing Instruments, Sissach, Switzerland), measuring the amount of scattered light in the mirror angle of the incident light, under an angle of 60° and recorded with *GlossTools* 1.7 software. Gloss was measured relative to a standard and given in gloss units (GU). All measurements were compared to a standard of black cardboard (HKS97N; standardized colour paper of the HKS-N-series; Hostmann-Steinberg K+E Druckfarben, H. Schmincke & Co., Germany) instead of the accompanied calibration standard of black polished glass. Since the normal use of this glossmeter is for highly glossy materials like car coatings, this procedure was done to achieve gloss data more widely distributed over the range of values provided by the glossmeter and therefore to examine differences in gloss between flowers more accurately. The black cardboard showed gloss of 1.74±0.28GU (n = 10) if measured with the manufactory standard. The particular flower parts were removed from flowers and positioned as flat as possible on black cardboard. For a more detailed insight into the technique of measurements with glossmeter see [15]. For each flower part of all investigated plant species, floral gloss values are included in Text S1. Gloss measurements were taken in the same direction of the flower petals as was used for transverse sections.

### Reflectance measurements and calculation of colour parameters

Reflectance measurements were performed with an USB4000 spectrophotometer (Ocean Optics, Inc., Ostfildern, Germany) and illumination was provided by a DH-2000-BAL light-source (Ocean Optics, Inc., Ostfildern, Germany), both connected via a coaxial fibre cable (QR400-7-UV-VIS, Ocean Optics, Inc., Dunedin, FL, USA). Since the values for photoreceptor excitation are not angle-dependent [14], all measurements were taken in an angle of 45° to the measuring spot with a pellet of barium sulphate used as white standard and a black film can used as black standard. Reflectance measurements were taken in the same direction towards flower petals than transverse sections.

As there is only little known about colour preferences of flower-visiting birds and even evidence that birds do not have spontaneous preferences for any colour or specific colour parameters, we evaluated the impact of epidermal cell shape on floral colouration for bees only. For this purpose, we calculated several colour parameters relevant for honeybees' response to colour cues [33], i.e. colour contrast to the background (we used an average spectrum of several green leaves), bee-subjective spectral purity, green contrast, and intensity. These colour parameters are thought to affect the foraging behaviour of bees and determine preferences [25]. The honeybee serves as an example for several trichromatic hymenopteran species with similar photoreceptor sensitivities in the ultraviolet, blue and green wavelength parts [22]. Colour contrast to the background was calculated using two colour vision models, i.e. the colour hexagon [34] and the receptor-noise limited model [35], both aligned to the colour vision system of hymenopterans. Bee-subjective spectral purity was calculated according to the colour hexagon model [34], [36], [37]. Green contrast, chroma and intensity were calculated independent of colour vision models [37], [38].

For exact calculations of colour parameters see [36]. All colour parameters of the investigated flower parts of all plant species are summarized in the Text S1.

### Statistical analysis

Correlations between epidermal cell shape, floral gloss, and colour parameters were analysed using Spearman rho correlations. To compare epidermal cell shape, floral gloss and all investigated colour parameters between each two flower parts of different pollinators we used one-way analysis of variance (ANOVA) with Tukey HSD as post-hoc test. Data were logarithm transformed to meet the assumption of variance homogeneity for the ANOVA. All statistical analyses were performed with the statistical computing software R 3.0.2 [39].

## Results

**Figure 2:**
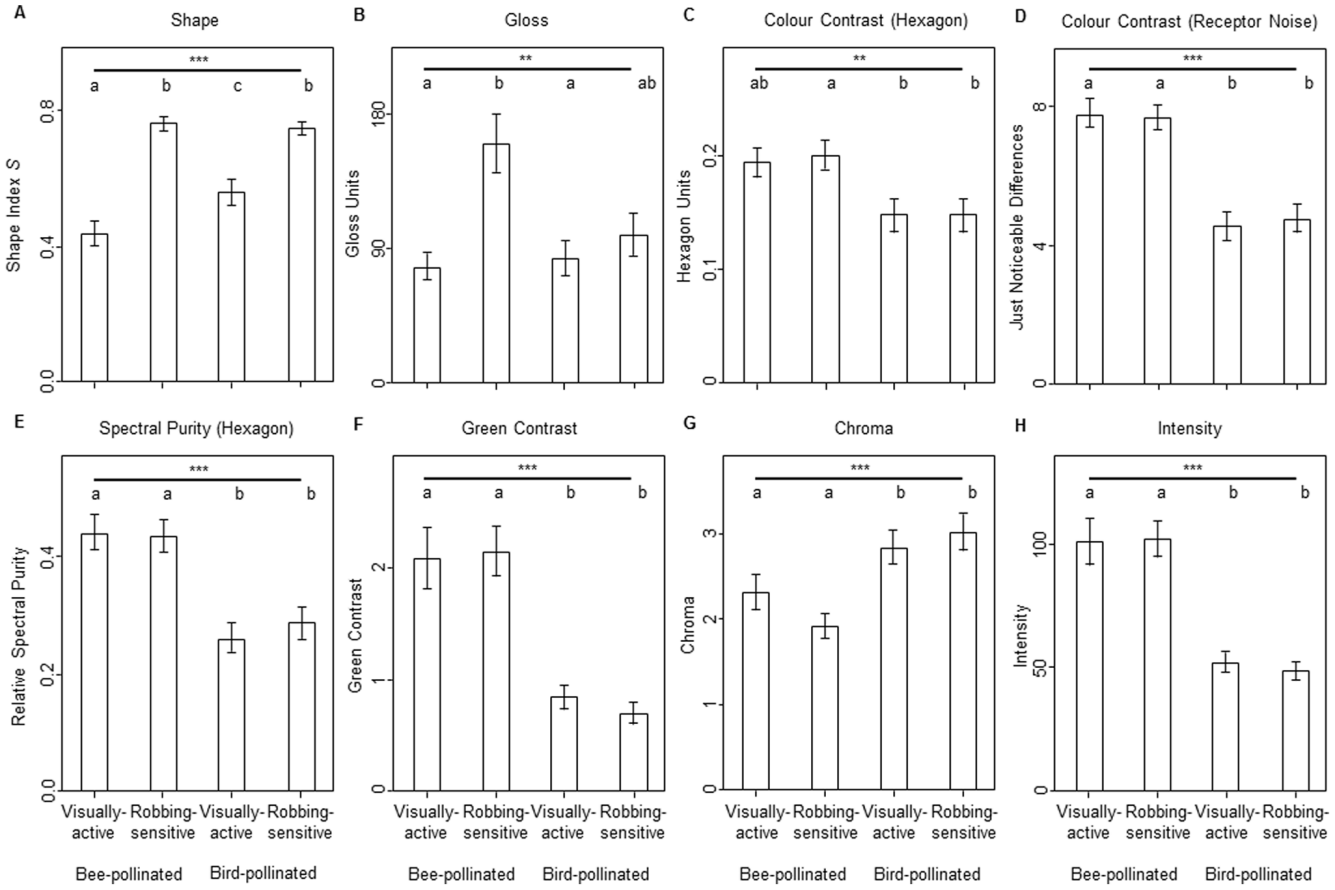
Epidermal cell shape, gloss and colour parameters of bee- and bird-pollinated flower parts. Means and standard errors of A) epidermal cell shape, B) floral gloss, colour contrast to the background in C) the colour hexagon model and D) in the receptor-noise limited model,(E) bee-subjective spectral purity according to the colour hexagon model, F) green contrast, G) chroma, and H) intensity for visually-active and for robbing-sensitive flower parts of bee- and bird-pollinated flowers. Asterisks above the bold line indicate differences according to one-way analysis of variance (ANOVA) with significance levels of ** for p<0.01 and *** for p<0.001. Different letters below the bold line denote significant differences according to pairwise comparisons using Tukey HSD.

Differences between cell shape, floral gloss, and colour parameters in relation to pollinators were found; main pollinator (i.e. bee or bird) as well as flower part (i.e. visually-active or robbing-sensitive parts) had a significant effect on shape, floral gloss as well as all investigated colour parameters (Figure 2).

Visually-active parts of bee-pollinated flowers had more often conical epidermal cells (corresponding to lower *S*-values), whereas robbing-sensitive parts of bee- as well as both parts of bird-pollinated flowers had more often flat epidermal cells (larger *S*-values) (Figs. 1, 2A). Visually-active parts of bird-pollinated flowers had more often intermediate values for S, and therefore convex or intermediate formed epidermal cells (Figures 1, 2A). Floral gloss was minimal in visually-active parts of bee-pollinated flowers, but did not significantly differ from both parts of bird-pollinated flowers (Figure 2B). Colour contrast to the background in the colour hexagon model, spectral purity in the hexagon model, green contrast, and intensity were larger for bee-pollinated flowers as compared to bird-pollinated ones, independent of the flower part (Figure 2C, E–F, H); however pairwise post-hoc comparisons for colour contrasts to the background in the colour hexagon model were not significant (Figure 2C). In contrast, colour contrast to the background in the receptor-noise limited model as well as chroma tended to be larger for both parts of bird-pollinated as compared to bee-pollinated flowers, but pairwise comparisons were not all significant (Figure 2D, G).

In the flowers studied there was a positive correlation between epidermal cell shape *S* and floral gloss, but all other correlations between *S* and the investigated colour parameters, as well as between floral gloss and the latter ones were not significant (Figure S1). As an exception, there was a significant positive correlation between floral gloss and bee-subjective spectral purity, (Figure S1). Moreover, we found significant correlations between specific colour parameters (Figure S1). However, due to the mathematical background and physiological conditions co-linearity between some colour parameter are given. For example, bee-subjective spectral purity in the hexagon model and colour contrast to the background in the colour hexagon model result both from the perceptual distance to the background in a rather similar manner.

## Discussion

A first survey of epidermal cell structure in angiosperms revealed that 79% of the investigated plant species show some form of papillate or conical epidermal cells [11]. The current study provides more differentiated results and demonstrates that the distribution of epidermal cell shape is largely explained by the effective pollinator as well as by their position on petals. Considering visually-active flower-parts among the 58 species studied, conical epidermal cells are more common in bee-pollinated flowers, whereas bird-pollinated flowers have more often flat epidermal cells. In contrast, flower parts which are vulnerable to nectar robbing are more often flat in bee- as well as in bird-pollinated flowers.

Both, the correlation between epidermal cell shape and pollinator guild as well as the within-flower patterns suggest that epidermal cell structure may play a significant role in determining flower-visitor choices.

### Effects of epidermal cell shape on colour sensation for bees and birds

Other than expected, the investigated colour parameters did not correlate with epidermal cell shape. Bee-pollinated flowers entirely appear of higher investigated colour parameters (i.e. bee-subjective spectral purity, chromatic contrast to the background, green contrast, chroma and intensity) as compared to bird-pollinated ones, irrespective of their epidermal cell shape. Thus, the tested colour parameters are not determined by means of cell shape only, provided that flower pigment concentration is similar in visually-active and robbing-sensitive flower parts. That the colouration of bird-pollinated flowers act as sensorial filter has been demonstrated previously, with comparably lower spectral purities and lower chromatic contrasts to the background as compared to bee-pollinated flowers of the same colour [2]. However, the light focusing effect of conical epidermal cells may enhance colour parameters like spectral purity only, if pigments are located properly within the epidermal cells [5].

These results are consistent with the work from Dyer *et al.* [40], who found no significant differences in the detectability for naïve bumblebees between wild-type and mutant flowers of *Anthirrinum majus*. However, Glover & Martin [41] as well as Comba *et al.* [16] showed that wild type flowers with conical epidermal cells received a higher frequency of approaches by bumblebees and yielded also a higher reproductive success as compared to flat-celled mutants.

Furthermore, epidermal cell shape does correlate with the amount of floral gloss. On epidermal surfaces with conically shaped cells, floral gloss appears only at the small apical tips of epidermal cells, producing a pattern of regularly arranged angle-dependent highlights on the petal. This is the case in bee-pollinated flowers, and the bright flashes arising from floral gloss at mirror geometry can act as an attractant for insects [14].

Other than expected, epidermal cell shape in bird-pollinated flowers does not predict floral gloss in a way that it fits to theoretical predictions. In fact, epidermal cell shape is only one factor among others determining gloss, and low glossiness might be caused by additional devices on (flat) epidermal cells like trichomes, surface-active residues, or surface micro-textures: these devices have been reported to decrease the amount of gloss on otherwise flat surfaces [13], [42].

But why do bird-pollinated flowers have flat epidermal cells at all, when it takes additional effort to reduce their intrinsic glossiness? Birds are fast flying flower visitors in habitats with adverse or alternating light conditions. Thus, on the one hand, dynamic visual displays in terms of floral gloss might improve the attention of birds, as plants might additionally exploit a pre-existing sensory bias for sparkling objects in birds [43]. However, flower-visiting birds, especially hummingbirds, need reliable floral cues operating on sunny as well as on cloudy and rainy days, because they need to feed on nectar more regular than bees. Thus the lacking invariability of gloss as visual cue in different ambient light conditions might foster a reduced glossiness of bird-pollinated flowers with flat surfaces.

The investigation of colour parameters and floral gloss in dependence on the epidermal cell shape suggest that the sensorial-floral-filter hypothesis does not apply. In fact, in our data set conical epidermal cells are restricted to visually-active parts of bee-pollinated flowers, but do not enhance the colour signal for bees.

### Effects of epidermal cell shape on floral grip for bees and birds

In contrast to hovering hummingbirds and perching flower-visiting birds, bees need to land on flowers while consuming rewards and thus need micro-textural surface structures for floral grip [19]. Thus, the results suggest that our second hypothesis applies, as we found the predicted distribution of conical epidermal cells in bee and flat epidermal cells in bird-pollinated flowers. Bumblebees are able to tactile discriminate between conical-celled flowers and flat-celled flowers, and prefer those flowers with rough surfaces, especially if the flowers are difficult to handle [19], [21] (but see [44]). Moreover, colour produced by differing surface properties can be used by bees as a cue to visually discriminate against flowers which lack grip [19]. Thus, even though bees do not show innate preferences for the colours produced by conical epidermal cells, bees might use slightly differences in their colouration for discrimination before landing. However, the results indicate that bees are not able to distinguish the colours produced by conical and flat epidermal cells.

### Effects of epidermal cell shape on floral grip for nectar robbers

Considering our dataset of flower parts which are vulnerable to nectar robbers, bee- as well as bird-pollinated flowers have more often flat surfaces consisting of flat epidermal cells. Therefore the third hypothesis applies, and flat surfaces might constitute a mechanical filter, hampering floral grip for nectar-robbing bees.

Surfaces consisting of flat epidermal cell surfaces make flowers difficult to handle for bees due to their slipperiness, and hamper bees to rest while consuming nectar [19]. The limitation of nectar robbing through a mechanical filter by flat surfaces might enhance the plants' reproductive success [28], [29].

Beside floral colouration and grip, several other flower traits are affected by epidermal cell shape, possessing bi-functionality for bees and birds as pollinators: Conical epidermal cells as compared to flat ones are suited to increase floral temperature used as floral reward by some insect visitors [16], (but see also [45]), but less important for homoiothermic birds. Beside this, conical as compared to flat epidermal cells improve overall flower size by influencing corolla reflexing abilities [6], and thereby promoting the detectability of flowers for bees [46]. Flower texture in form of conical epidermal cells acts additionally as tactile cue after landing and guide the bee towards the reward [18]. All these flower traits have different meanings for bees and birds, with conical epidermal cells more suitable in bee-pollinated flowers and flat epidermal cells more suitable for bird-pollinated ones. Bird-pollinated plants evolve mainly from bee-pollinated ancestors ([47], and references within). Therefore an evolutionary shift from conical epidermal cells in bee-pollinated ancestors towards bird-pollinated plants with a derived flat epidermal surface structure is conceivable. The evolutionary shift includes adaptations towards birds but at the same time maladaptations for bees, as was shown already for several other flower traits [48], [49].

The current study shows for the first time that epidermal cell shape is pollinator and flower part dependent. This ambiguity provides, together with experimental studies, evidence that epidermal cell shape is a multi-functional adaptive floral trait affecting grip required for bees as well as the floral colour signal as one of the most selective floral attractants. Petals' surface structure might be an important, but hitherto neglected, flower trait structuring the visitor composition of flowers and should be considered in plant-pollinator and plant-antagonist networks.

## Supporting Information

Figure S1 Heatmap visualizing the results of Spearman's rho correlation between epidermal cell shape *S*, floral gloss, and all investigated colour parameters. The significance level of correlation is colour-coded with black for p<0.001, dark grey for p<0.05, light grey for p>0.05, and white for p>0.1. In the upper triangle of the symmetric matrix, rho-values are given. In the lower triangle, corresponding scatter plots are presented.
(TIF)

Text S1 Excel file including a list of studied plant species with literature reference about pollination mode, measured flower parts, floral colour, cell shape parameters and shape index *S*, floral gloss values and colour parameters.
(XLSX)
